# Feasibility of Outpatient Stem Cell Transplantation in Multiple Myeloma and Risk Factors Predictive of Hospital Admission

**DOI:** 10.3390/jcm11061640

**Published:** 2022-03-16

**Authors:** Kristin Larsen, Horace Spencer, Meera Mohan, Clyde Bailey, Kerri Hill, Mathew Kottarathara, Richa Parikh, Shadiqul Hoque, Amani Erra, Angel A. Mitma, Pankaj Mathur, Lakshmi Yarlagadda, Sravani Gundarlapalli, Yetunde Ogunsesan, Munawwar Hussain, Nishanth Thalambedu, Jaskirat Sehti, Samer Al Hadidi, Sharmilan Thanendrarajan, Monica Graziutti, Maurizio Zangari, Bart Barlogie, Frits van Rhee, Guido Tricot, Carolina Schinke

**Affiliations:** 1 Myeloma Center, University of Arkansas for Medical Sciences, Little Rock, AR 72205, USA; knlarsen@uams.edu (K.L.); baileyclyde@uams.edu (C.B.); hillkerrim@uams.edu (K.H.); mkottarathara@uams.edu (M.K.); richaparikh36@uams.edu (R.P.); shoque@uams.edu (S.H.); aerra@uams.edu (A.E.); amitma@uams.edu (A.A.M.); pmathur@uams.edu (P.M.); lyarlagadda@uams.edu (L.Y.); sgundarlapalli@uams.edu (S.G.); yogunsesan@uams.edu (Y.O.); mhussain@uams.edu (M.H.); nthalambedu@uams.edu (N.T.); jsethi@uams.edu (J.S.); salhadidi@uams.edu (S.A.H.); sthanendrarajan@uams.edu (S.T.); graziuttim@uams.edu (M.G.); mzangari@uams.edu (M.Z.); blackbarlogie@gmail.com (B.B.); vanrheefrits@uams.edu (F.v.R.); gtricot@uams.edu (G.T.); 2 Department of Biostatistics, University of Arkansas for Medical Sciences, Little Rock, AR 72205, USA; spencerhoracej@uams.edu; 3 Medical College of Wisconsin Cancer Center, Froedtert Hospital, Milwaukee, WI 53226, USA; memohan@mcw.edu

**Keywords:** multiple myeloma, autologous stem cell transplantation, hospitalization

## Abstract

High-dose chemotherapy followed by autologous stem cell transplantation (ASCT) remains the standard of care for multiple myeloma (MM) patients. Although outpatient ASCT has been shown to be safe and feasible, the procedure is overall rare with most patients in the US undergoing inpatient ASCT. Furthermore, hospitalization rates for patients that undergo outpatient ASCT remain high. Adequate markers that predict hospitalization during outpatient ASCT are lacking, yet would be of great clinical value to select patients that are suited to outpatient ASCT. In this study we aimed to elucidate differences between planned outpatient and inpatient ASCT and further evaluated clinical characteristics that are significantly associated with hospitalization during planned outpatient hospitalization. Factors that were significantly associated with a planned inpatient ASCT included an advanced MM disease stage, worse performance status as well as non-Caucasian race, while low albumin levels and female gender were significantly associated with hospitalization during outpatient ASCT. The results of this analysis provide crucial knowledge of factors that are associated with planned inpatient ASCT and hospitalization during outpatient ASCT and could guide the treating physician in decision-making and further facilitate outpatient transplantation.

## 1. Introduction

High-dose chemotherapy followed by autologous stem cell transplant (ASCT) remains the standard treatment for eligible newly diagnosed multiple myeloma (MM) [1,2] and also has shown significant efficiency in relapsed MM patients [3,4]. While historically, due to logistic issues and concerns regarding toxicities and infections most ASCTs were performed in an inpatient setting, the swift recovery after peripheral ASCT and improvements in supportive care have enabled patients to receive ASCTs as outpatients [5]. Previous studies have demonstrated that outpatient ASCTs are not only safe and feasible, but also associated with reduced healthcare costs and better outcomes compared to inpatient settings [6,7,8]. Yet, the percentage of outpatient ASCTs remains relatively small and there is also a high rate of hospital admissions, which in some reports is as high as 55% [9]. Meticulous patient selection is the key to successful outpatient ASCT and several studies have reported that patients undergoing inpatient ASCTs tend to be older in age and have worse performance status compared to those that start their ASCTs as outpatients [10,11,12]. However, other factors that are associated with inpatient ASCTs have not been explored systematically to date. Furthermore, risk factors indicating whether a patient who started as an outpatient ASCT will require later hospital admission have yet to be elucidated and would be of tremendous clinical value.

The University of Arkansas for Medical Sciences (UAMS) started performing outpatient ASCTs for MM in 1995 and has since performed 10,320 ASCTs with 6690 of these being performed on an outpatient basis. To account for most recent supportive care regimen and antibiotic treatments, we have limited our analysis to patients receiving ASCT since 2015 and summarize in this report our experience in performing 1165 ASCTs, of which 745 were outpatient, in 811 consecutive MM patients from 2015–2019. Patient characteristics and clinical outcomes between in- and outpatients were compared and prognostic factors that predict for inpatient status at ASCT initiation or admission during outpatient transplantation were explored.

## 2. Methods

### 2.1. Patients

We performed a retrospective chart review of all MM patients undergoing ASCT between 2015 and 2019 and separated patients who had their first ASCT performed in this time frame from those who received a second ASCT to avoid confounders and bias. Within this time frame, 811 patients received a first ASCT, which was followed by a second ASCT in 354 patients. Of note is that patients who had two ASCTs, 96% (341/354) were performed as tandem ASCTs (within 6 months of first ASCT) while 4% (13/354) were done as salvage ASCTs for relapsed MM. Data on first and second ASCTs were analyzed separately to allow for accurate investigation of transplant outcomes and factors that influenced hospital admission. Within each transplant cohort (1st vs. 2nd ASCT), patients were divided into 3 groups: those who received their ASCT as inpatients (group 1) vs. patients that started their ASCT as outpatients, but required hospital admission within 20 days of stem cell infusion (Hosp, group 2) vs. patients who had their ASCT entirely as outpatients (No Hosp, group 3).

Patients were eligible for ASCT provided their organ functions were adequate, as determined by cardiac ejection fraction > 40%, forced vital capacity (FVC) and CO diffusion capacity ≥ 50%, liver function abnormalities less than twice the upper range of normal, and creatinine ≤ 3 mg/dL. The decision to proceed with outpatient stem cell transplant was made by the treating oncologist and although there were no official departmental criteria to determine the choice of treatment plan, consensus criteria were generally applied [13]. Patients needed an adequate performance status (Karnofsky ≥ 80), normal organ function, negative pre-transplant infectious screening (CRP measurement, imaging, and additional tests as clinically indicated), a committed caregiver, and to be housed within 45 min of UAMS to be considered for outpatient ASCT.

### 2.2. High-Dose Chemotherapy Conditioning Regimen

The preparative regimen for ASCT was divided into 3 groups: (1) melphalan-based therapy with patients receiving either 200 mg/m^2^ or 140 mg/m^2^ of Melphalan, (2) BEAM-based therapy (carmustine, etoposide, cytarabine melphalan), or (3) VDT-PACE (bortezomib, dexamethasone, thalidomide, cisplatin, adriamycin, cyclophosphamide and etoposide) with low-dose melphalan (80 mg/m^2^)-based therapy (also called hybrid regimen). The choice of a myeloablative regimen was made by the treating oncologist and was decided upon careful consideration of age, performance status, and organ function. 

### 2.3. Supportive Care

Prior to conditioning, all patients had a central catheter placed into the subclavian or jugular vein. All patients were treated with prophylactic doses of levofloxacin, acyclovir and fluconazole. Filgastrim at 5 mg/kg was started daily in all patients either at day +5 or if the leucocyte count dropped below 2000/µL.

All outpatients were required to have an adult caregiver during the entire transplant course. Patients were evaluated daily by an experienced transplant nurse practitioner. Laboratory studies were performed daily and, if necessary, intravenous (iv) hydration, electrolyte replacement or blood product transfusion were administered. All patients were instructed in catheter care, temperature monitoring, and use of infusers. The clinic was open 7 days a week from 7 am to 7 pm. After hours, the patients had access to a transplant physician for medical emergencies. All patients were given an infuser bag containing IV cefepime that was to be administered immediately in the case of febrile episodes to prevent delay of treatment for possible sepsis. 

### 2.4. Engraftment Criteria

Time to neutrophil engraftment was defined as the first day of absolute neutrophil count (ANC) > 500/µL for 3 consecutive days. Time to platelet recovery was defined as the first day of platelet count > 20,000/µL without transfusion support for at least 7 days.

### 2.5. Criteria for Hospital Admission and Transplant Related Mortality

The reason for admission of patients who started their ASCT process as outpatients was: (1) infections and neutropenic fever, (2) intractable nausea/diarrhea/poor oral intake, (3) cardiac events, and (4) other. Non-relapse-related mortality was assessed at 30 and 100 days and defined as death not due to disease progression.

### 2.6. Statistical Analysis

The data were divided into two sets according to transplant number (ASCT 1 and 2). Three hospitalization-related endpoints were analyzed for each set of data: (1) inpatient status, (2) any hospitalization; and (3) outpatient hospitalizations. Multivariate logistic regression models were used to assess factors that may be associated with each of the endpoints (R version 4.1.2 [R Foundation for Statistical Computing, Vienna, Austria. URL https://www.R-project.org/]). The following variables appear in all of the models: age (∆ = 5 yrs), gender (referent: males), race—Not White (referent: Caucasian), BMI (∆ = 5 kg/m^2^), karnofsky 80 (referent: 90 and unknown), chemotherapy—hybrid and other (referent: melphalan), albumin (∆ = 0.5 g/dL), β2-Microglobulin (∆ = 2 µg/mL), creatinine (∆ = 0.5 µmol/mL) and hemoglobin (∆ = 1.25 g/dL). Risk was defined by the GEP70 classifier as previously reported [14]. Other variables were included based on the dataset or endpoint being analyzed. These exceptions follow: The analysis of the 2nd transplant data includes a variable representing whether the patient was hospitalized during their initial transplant (referent: no prior hospitalization). Models of outpatient hospitalizations include total CD34 cells transplanted (∆ = 2 × 106 cells). Conditional inference classification trees were used to identify subgroups of patients with low rates of hospitalizations for each dataset described above.

## 3. Results

### 3.1. Patient Characteristics and Parameters for Inpatient ASCT

To allow for unbiased analysis, we examined patients that underwent their first and second ASCT separately. Of the 811 patients with first ASCT, 61.7% (500/811) of patients initiated their ASCT as outpatients, Table 1. Of those, 31.6% (158/500) required hospital admission during their treatment course (within 20 days of ASCT, Group 2), while 68.4% (342/500) patients were able to complete their ASCT on an outpatient basis (Group 3). Similarly, the majority of patients (69.5%, 246/354) initiated their second ASCT as outpatients with 28% (69/246) requiring hospital admission, Supplementary Table S1. Of note is that patients who had two ASCTs, 96% (341/354) were performed as tandem ASCTs (within 6 months of first ASCT) while 4% (13/354) were done as salvage ASCTs for relapsed MM. Median age was 61 years (range: 29–80) for first ASCT with no significant differences between patients who received an ASCT as inpatients (Group 1), required hospitalization during outpatient ASCT (Group 2), or completed the procedure as outpatients (Group 3) and 57 years (range: 29–73) for the second ASCT (n = 354), indicating that predominantly younger patients proceeded to a second ASCT. 

**Figure 1:**
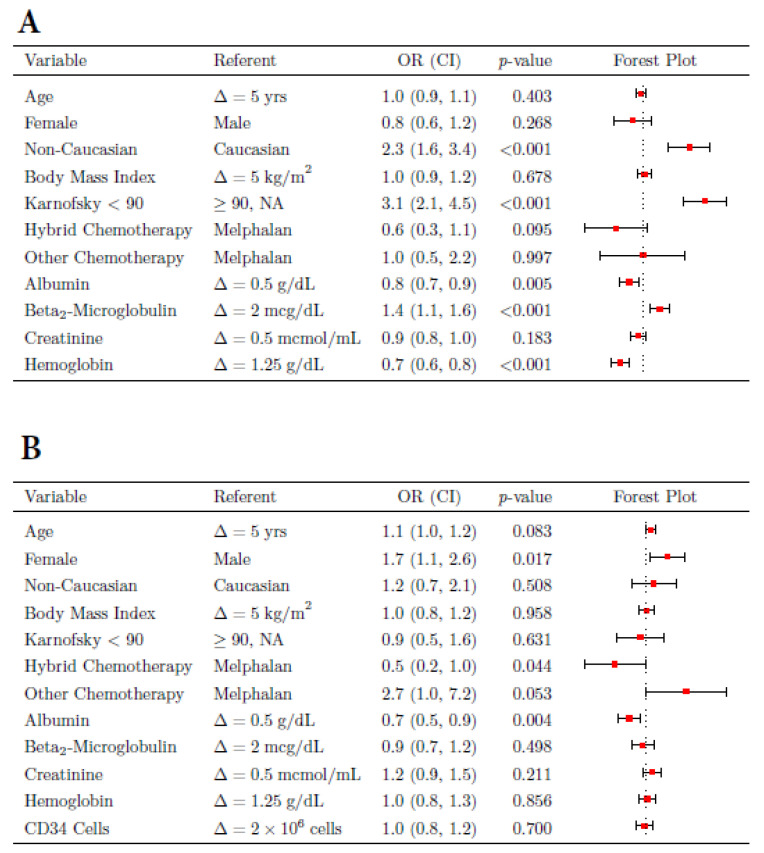
Clinical parameters associated with inpatient status (Group 1) for first ASCT (**A**). Variables associated with hospitalization in patients who started their first ASCT as outpatients (Groups 2 and 3) (**B**). OR = odds ratio, CI = confidence interval.

We then aimed to identify parameters that were associated with inpatient status (Group 1) compared to patients that initiated treatment on an outpatient basis (Groups 2 and 3), Figure 1A and Supplementary Figure S1. For the first ASCT a worse performance status (Karnosfsky < 90, ORR = 3.06, *p* < 0001) was most significantly associated with inpatient status. Furthermore, factors directly linked to advanced MM disease, including lower albumin (ORR = 1.28, *p* < 0.01), higher b-2-microglobulin (ORR = 1.35, *p* < 0.001), and a lower hemoglobin (ORR = 1.51, *p* < 0.0001) were significantly associated with inpatient ASCT. Interestingly, we also found that patients of non-Caucasian race, 82% being African Americans (AA), had a significantly higher risk of proceeding with inpatient ASCT, ORR = 2.31, *p* ≤ 0.001. The reason for this observation is not quite clear, however we observed that patients of non-Caucasian race undergoing first ASCT had worse Karnofsky performance status (*p* ≤ 0.001), worse albumin (*p* ≤ 0.01), and higher beta-2-microglobulin (*p* ≤ 0.01) compared with non-Caucasian patients who initiated ASCT as an outpatient (data not shown), suggesting that the higher proportion of non-Caucasian patients undergoing inpatient ASCT was mainly based on patient and disease status rather than socioeconomic factors. Interestingly, other parameters such as age, body mass index (BMI), and chemo regimen utilized for conditioning were of no significance in this analysis.

Next, we examined factors that were significantly associated with inpatient status during second ASCT and show that a previous history of any hospitalization (Group 1 or 2) was the most significant parameter associated with inpatient status, ORR = 32.48, *p* < 0.0001, Supplementary Figure S1. Furthermore, poor performance status (Karnofsky < 90, ORR = 2.66, *p* < 0.05), non-Caucasian race (ORR = 3.93, *p* < 0.001), and low hemoglobin (ORR = 1.69, *p* < 0.01) were all significantly associated with inpatient status at 2nd ASCT. 

### 3.2. Factors Contributing to Hospital Admission during Outpatient ASCT

Of the 500 patients that started their first ASCT as outpatients, 31.6% (158/500) required admission during their treatment course (within 20 days of ASCT) and 28% (69/246) of patients were admitted during second ASCT. The main reasons for hospital admission of patients undergoing first and second ASCTs were infections/neutropenic fever (52% vs. 61%) and intractable nausea/diarrhea/poor oral intake (34% vs. 31%), followed by other events such as arrhythmia and cardiovascular events (7% vs. 6%), MM-related pain (4% vs. 2%) and bleeding or risk of bleeding (3% vs. not observed), Table 2. Median day of admission was day 7.7 (range 1.6–15.7) post-ASCT with a median length of hospital stay of 8 (range 2–49) days for first ASCT and day 7.7 (range 1.6–14.8) and 9 days (range (2–19) for the second ASCT. 

We then determined variables that would predict for hospitalization during outpatient ASCT in 1st and 2nd ASCT (Figure 1B and Supplementary Figure S2). For 1st ASCT we identified low albumin levels (ORR = 1.51, *p* ≤ 0.01), and interestingly, female gender (ORR = 1.69, *p* ≤ 0.05), as variables associated with higher risk of admission, while the use of hybrid ASCT (ORR= 0.46, *p* ≤ 0.05)—a less toxic regimen compared to melphalan or BEAM conditioning—was associated with a minor risk of admission. Higher age and the use of BEAM conditioning substantially increased the risk of hospitalization, but were not quite significant variables. For patients proceeding to a 2nd ASCT in an outpatient setting, the main predictive parameters associated with hospitalization were a worse performance status (Karnofsky < 90, ORR = 3.17, *p* = 0.01) and a history of hospitalization (either Group 1 or 2) during first ASCT, ORR = 3.12, *p* ≤ 0.001.

### 3.3. Engraftment and Non-Relapse Mortality

We further investigated differences in transplant-related outcomes between these groups, in particular variation between engraftment and non-relapse mortality (NRM) after ASCT (Supplementary Tables S2 and S3). Neutrophil engraftment was mildly, but significantly, delayed in the inpatient group compared to those patients that started as outpatients (Groups 2 and 3), which was seen in first (*p* < 0.001) and second ASCT (*p* = 0.03). Platelet engraftment was interestingly slower in the outpatient groups (Groups 2 and 3) in both ASCT cohorts compared to the inpatient group, albeit not quite significant. The reason for this could be the prompt discharge of outpatients back to their local oncologists, which occurs after neutrophil engraftment but not necessarily after platelet engraftment (platelet count ≥ 20,000 without transfusion support for 7 days), indicating that platelet engraftment will only be recorded after the patient’s return visit to UAMS, which usually happens after 4–12 weeks.

NRM at 30 and 100 days for the entire transplant cohort was 0.5% at 30 days and 2.9% at 100 days for first ASCT and 0.8% and 3.1% for second ASCT. While the 30-day NRM did not differ significantly between the inpatient and outpatient groups for either ASCT, the 100 NRM was significantly higher in patients who initiated their ASCT as inpatients (Group 1) compared to outpatients (Groups 2 and 3) for first ASCT (*p* < 0.002) and 2nd ASCT (*p* = 0.04) (Supplementary Tables S2 and S3). These results corroborate previous findings [12] and are not surprising since a worse performance status and compromised organ function have been associated with worse outcome in ASCT patients [15,16]. 

## 4. Discussion

Outpatient transplantation for the treatment of MM has become increasingly common because of the introduction of peripheral ASCT and advancements in supportive care. Yet, in most academic centers the percentage of patients who receive transplantation on an outpatient compared to an inpatient basis remains quite low. Here we present our results on one of the largest reported outpatient ASCT populations to date. In line with previous studies, we show that patients who received ambulatory ASCT tended to be more physically fit at baseline [9,10,16]. We also show a racial difference with significantly more non-Caucasians undergoing inpatient ASCT. The reason for this observation is not entirely clear but is unlikely solely due to socioeconomic reasons. 

Non-relapse mortality was significantly higher and 2-year overall survival rates significantly lower in the inpatient ASCT group in line with previous findings [12]. This is not surprising since a worse performance status and compromised organ function have been associated with worse outcomes in ASCT patients [15,16]. Of those patients who initiated their ASCT in the outpatient setting (Groups 2 and 3), 31% required hospital admission. This is a smaller fraction than what has been reported in the past and may reflect an improved supportive care regimen, in particular anti-emetic strategies, in the ambulatory setting [17,18]. The reasons for hospital admission for the vast majority of patients were either infection/sepsis or intractable nausea/vomiting or diarrhea. Our findings demonstrate that older age, low serum albumin, and female gender were significant and independent predictors of hospital admission for those patients who initiated their first ASCT in the ambulatory setting. Older age is associated with decreased physical fitness and increased comorbidities, while low serum albumin serves as a proxy for a worse nutritional status, inflammatory status, and advanced MM stage. The association between being female and greater likelihood of hospital admission was unexpected. While much research on gender-specific outcomes in cancer diseases has been conducted over recent decades, it is only recently that differences in gender-specific side effect profiles have been acknowledged. A recent study in advanced esophagogastric cancer has shown that women have a significantly higher incidence of nausea, vomiting, and diarrhea compared to men [19]. The reasons for that remain unexplored, but one explanation could be related to differences in metabolism between females and males. Interestingly, female gender and low albumin were not significant parameters for hospital admission during the second ASCT, but only a history of prior hospitalization and poor performance status were significant. 

It is of note that while we have identified factors that are significantly associated with an inpatient status or hospitalization after transplantation, the evaluation of some other possible relevant clinical parameters was limited due to the low percentage of available data. This is particularly true for risk status and response to induction therapy prior to ASCT. Interestingly, some parameters that have been historically associated with worse outcomes, such as older age or higher BMI, [20,21] were not significantly associated with inpatient ASCT or hospitalization during outpatient ASCT in our analysis. The reasons for this might be that doses of the chemo conditioning regimen are typically adjusted for age and have hence become more tolerable also for the elderly population. Obesity is a factor known to increase MM risk, yet its effect on outcomes after treatment initiation is controversial [21,22]. Here, we did not find a high BMI to be a significant factor for inpatient status or hospitalization rates; however, morbidly obese patients tend to have decreased organ function and worse performance status and are typically not good transplant candidates in the first place, suggesting that these patients were excluded from the study.

## 5. Conclusions

Overall, we present compelling data that underscore the feasibility and safety of outpatient ASCT in MM. For patients that initiate their transplant on an outpatient basis, female gender and low albumin levels were clinical parameters that were significantly associated with a hospitalization during the transplant. Furthermore, the conditioning regimen played a crucial role and patients with more aggressive chemotherapies (such as BEAM) were at higher risk of hospitalization. The identification of risk factors predicting for hospital admission during outpatient ASCT are useful tools that could help guiding the treating physician and further facilitate outpatient transplantation.

## Figures and Tables

**Table 1:** Patient characteristics in first ASCT (*n* = 811). BMI = body mass index, GEP70 = gene expression 70 risk classifier, WBC = white blood count, ANC = absolute neutrophil count, DLCO = diffusion capacity for carbon monoxide, ECHO = echocardiogram, FEV1 = forced expiratory volume, VGPR = very good partial response.

Variable				Outpatient			
	I			Hosp.		o Hosp.	
	N	Statistic	N	Statistic	N	Statistic	*p*-Value
Age, median (yrs)	311	61.2 (9.4)	158	62.2 (8.7)	342	60.6 (9.4)	0.183
Female	311	45.3% (141)	158	50.6% (80)	342	40.6% (139)	0.103
African American	311	25.4% (79)	158	15.8% (25)	342	12.0% (41)	<0.001
Caucasian	311	69.1% (215)	158	82.3% (130)	342	84.8% (290)	<0.001
Other Race	311	5.5% (17)	158	1.9% (3)	342	3.2% (11)	0.137
BMI (kg/m^2^)	311	28.9 (6.6)	158	29.1 (5.9)	341	29.2 (5.5)	0.516
Karnofsky *<* 90	311	34.4% (107)	158	12.0% (19)	342	13.2% (45)	<0.001
Conditioning Regimen							
Melphalan	311	86.8% (270)	158	86.1% (136)	342	87.4% (299)	0.899
Hybrid Chemo	311	8.7% (27)	158	7.0% (11)	342	10.2% (35)	0.511
BEAM Chemo	311	4.5% (14)	158	7.0% (11)	342	2.3% (8)	0.046
Risk score (GEP 70)							
High Risk	101	29.7% (30)	70	24.3% (17)	162	22.8% (37)	0.469
Other parameters							
Albumin (g/dL)	311	3.6 (0.6)	158	3.7 (0.5)	341	3.8 (0.4)	<0.001
WBC	311	4.6 (2.3)	158	5.7 (3.0)	341	6.3 (3.4)	
ANC	303	3.3 (2.2)	154	4.8 (3.0)	335	5.2 (3.5)	<0.001
*β*2-M (µg/dL)	308	5.3 (6.2)	158	3.2 (2.0)	341	3.1 (1.9)	<0.001
Creatinine (µmol/mL)	311	1.3 (1.4)	158	1.0 (0.4)	341	1.0 (0.6)	0.513
Glucose (mg/dL)	311	119.8 (52.0)	158	128.2 (53.3)	341	123.9 (37.5)	<0.001
Hemoglobin (g/dL)	311	9.7 (1.4)	158	10.5 (1.2)	341	10.7 (1.5)	<0.001
Total CD34 (×10^6^ cells)	309	5.9 (1.8)	157	5.8 (1.8)	341	5.8 (1.8)	0.457
DLCO (%)	236	75.2 (14.6)	109	76.8 (14.4)	203	81.0 (14.0)	<0.001
ECHO (mm/Hg)	298	58.2 (5.6)	142	58.9 (4.1)	310	58.7 (4.4)	0.810
FEV1 (%)	216	83.3 (20.3)	101	90.8 (16.5)	259	93.2 (18.9)	<0.001
Response (≥VGPR)	161	66.5% (107)	86	67.4% (58)	184	67.4% (124)	0.991

**Table 2:** Reasons for hospitalization of patients that initiated their ASCT on an outpatient basis.

Reason for Admission	1st ASCT (*n* = 158)	2nd ASCT (*n* = 69)
Sepsis/infection	52% (*n* = 83)	61% (*n* = 41)
Intractable vomiting/diarrhea, poor oral intake, electrolyte imbalance	34% (*n* = 53)	31% (*n* = 21)
Arrhythmia (atrial fibrillation), cardiovascular events	7% (*n* = 12)	6% (*n* = 5)
Intractable pain	4% (*n* = 6)	2% (*n* = 1)
Bleeding, risk of bleeding	3% (*n* = 4)	Not observed

## Data Availability

The data presented in this study are available on request from the corresponding author.

## Supplementary Materials

The following supporting information can be downloaded at: https://www.mdpi.com/article/10.3390/jcm11061640/s1, Table S1: Patient characteristics- second transplant (N = 354); Table S2: Transplant related outcomes for 1st ASCT. NRM = non relapse mortality; Table S3: Transplant related outcomes for 2nd ASCT. NRM = non relapse mortality; Figure S1: Risk factors that predict for Inpatient Status (group 1) at time point of 2nd ASCT; Figure S2: Factors associated with hospitalization for patients who initiated their 2nd ASCT as outpatients.
