# Supplementary material for: Feasibility of Outpatient Stem Cell Transplantation in Multiple Myeloma and Risk Factors Predictive of Hospital Admission
Source: J Clin Med. 2022 Mar 16;11(6):1640. doi: 10.3390/jcm11061640 (PMC8955129; doi:10.3390/jcm11061640)

Table S1. Patient characteristics- second transplant (N=354)

	Inpatient		Outpatient				
			Hosp.		No Hosp.		
Variable	N	Statistic	N	Statistic	N	Statistic	P-value
Age, median (yrs)	108	57.5 (9.4)	69	58.8 (7.1)	177	57.1 (8.9)	0.436
Female	108	52.8% (57)	69	44.9% (31)	177	40.7% (72)	0.135
African American	108	33.3% (36)	69	17.4% (12)	177	10.2% (18)	<0.001
Caucasian	108	63.0% (68)	69	81.2% (56)	177	85.3% (151)	<0.001
Other Race	108	3.7% (4)	69	1.4% (1)	177	4.5% (8)	0.652
BMI (kg/m^2)	108	29.3 (7.1)	69	28.6 (6.0)	177	29.1 (5.7)	0.739
Karnofsky < 90	108	38.9% (42)	69	20.3% (14)	177	7.3% (13)	<0.001
Conditioning Regimen							
Melphalan	108	69.4% (75)	69	82.6% (57)	177	81.4% (144)	0.043
Hybrid Chemo	108	20.4% (22)	69	7.2% (5)	177	10.2% (18)	0.018
Other Chemo	108	10.2% (11)	69	10.1% (7)	177	8.5% (15)	0.859
Risk score (GEP 70)							
High Risk	36	44.4% (16)	21	42.9% (9)	103	20.4% (21)	0.007
Other parameters							
Albumin (g/dL)	108	3.6 (0.5)	69	3.8 (0.3)	177	3.8 (0.4)	0.006
WBC	108	4.3 (2.1)	69	6.4 (3.9)	177	5.9 (2.7)	<0.001
ANC-S	108	3.1 (2.0)	69	5.2 (3.9)	177	4.5 (2.7)	<0.001
β_2-M (μg/dL)	108	4.6 (5.3)	69	2.9 (1.5)	177	2.8 (1.6)	<0.001
Creatinine (μmol/mL)	108	1.3 (1.4)	69	1.0 (0.3)	177	0.9 (0.4)	0.093
Glucose (mg/dL)	108	116.1 (36.0)	69	122.2 (45.5)	177	116.8 (31.0)	0.557
Hemoglobin (g/dL)	108	9.8 (1.4)	69	11.0 (1.5)	177	11.1 (1.5)	<0.001
Total CD34 ($\times 10^6$ cells)	105	7.1 (2.3)	68	7.4 (2.3)	177	7.1 (2.0)	0.768
DLCO (%)	94	72.7 (14.3)	62	74.5 (14.0)	116	79.5 (15.0)	0.005
ECHO (mm/Hg)	106	57.5 (6.1)	68	57.7 (4.4)	156	58.5 (3.9)	0.427
FEV1 (%)	79	82.9 (20.1)	47	89.5 (17.5)	142	93.4 (17.6)	<0.001
Response	44	90.9% (40)	32	84.4% (27)	105	83.8% (88)	0.535
Prior Hosp?	108	90.7% (98)	69	37.7% (26)	177	16.9% (30)	<0.001

Table S2- Transplant related outcomes for 1st ASCT. NRM= non relapse mortality

	Inpatient (group 1)	Hospitalized (group 2)	No hospitalization (group 3)	p-value
**Neutrophil engraftment, mean days (range)**	11.63 (7-25)	11.14 (9-23)	11.09 (9-25)	<0.001
**Platelet engraftment mean days (range)**	18.23 (6-71)	17.35 (6-75)	20.7 (6-75)	0.07
**NRM 30 days**	0.3% (1/311)	1.2% (2/158)	0.3% (1/342)	NS
**NRM 100 days**	4.8% (15/311)	1.9% (3/158)	0.6% (2/342)	0.002

Table S3- Transplant related outcomes for 2nd ASCT. NRM= non relapse mortality

	**Inpatient (group 1)**	**Hospitalized (group 2)**	**No hospitalization (group 3)**	**p-value**
**Neutrophil engraftment, mean days (range)**	11.63 (7-25)	11.14 (9-23)	11.09 (9-25)	<0.001
**Platelet engraftment mean days (range)**	18.23 (6-71)	17.35 (6-75)	20.7 (6-75)	0.07
**NRM 30 days**	0.3% (1/311)	1.2% (2/158)	0.3% (1/342)	NS
**NRM 100 days**	4.8% (15/311)	1.9% (3/158)	0.6% (2/342)	0.002

Figure S1. Risk factors that predict for Inpatient Status (group 1) at time point of 2nd ASCT. Non-Caucasian race (p<0.001), worse Karnofsky Index (p=0.02) and a history of prior hospitalization during the 1st ASCT (p<0.0001) are significantly associated with an Inpatient Status.

Variable	Referent	OR (CI)	Forest Plot	p-value
Age	$\Delta = 5$ yrs	1.00 (0.80, 1.24)		0.98
Female	Male	0.74 (0.34, 1.58)		0.43
Non-Caucasian	Caucasian	3.93 (1.76, 8.79)		<0.001
Body Mass Index	$\Delta = 5$ kg/m^2	1.01 (0.78, 1.30)		0.93
Karnofsky < 90	≥ 90, NA	2.66 (1.20, 5.89)		0.02
Hybrid Chemotherapy	Melphalan	1.01 (0.36, 2.83)		0.98
Other Chemotherapy	Melphalan	0.58 (0.18, 1.86)		0.36
Albumin	$\Delta = 0.5$ g/dL	0.81 (0.55, 1.20)		0.29
β_2-Microglobulin	$\Delta = 2$ μg/dL	1.20 (0.76, 1.88)		0.43
Creatinine	$\Delta = 0.5$ μmol/mL	1.12 (0.72, 1.75)		0.61
Hemoglobin	$\Delta = 1.25$ g/dL	0.59 (0.41, 0.84)		0.003
Prior Hospitalization?	No Prior Hosp.	32.48 (14.42, 73.18)		<0.0001

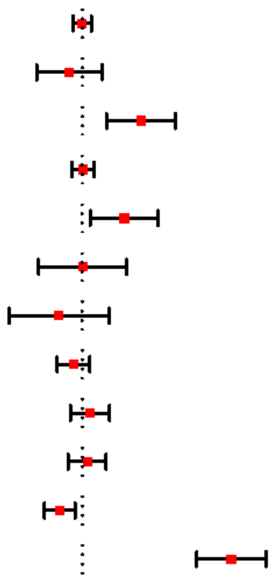

Figure S2. Factors associated with hospitalization for patients who initiated their 2nd ASCT as outpatients. A worse Karnofsky Index (p=0.01) and a history of prior hospitalizations during 1st ASCT (<0.001) are significantly associated with hospitalization during 2nd ASCT.

Variable	Referent	OR (CI)	Forest Plot	p-value
Age	$\Delta = 5$ yrs	1.16 (0.96, 1.41)		0.12
Female	Male	1.32 (0.62, 2.80)		0.47
Non-Caucasian	Caucasian	1.74 (0.74, 4.07)		0.20
Body Mass Index	$\Delta = 5$ kg/m^2	0.91 (0.70, 1.20)		0.51
Karnofsky < 90	≥ 90, NA	3.17 (1.32, 7.64)		0.01
Hybrid Chemotherapy	Melphalan	0.49 (0.15, 1.64)		0.25
Other Chemotherapy	Melphalan	1.30 (0.42, 4.02)		0.65
Albumin	$\Delta = 0.5$ g/dL	0.84 (0.54, 1.30)		0.43
β_2-Microglobulin	$\Delta = 2$ μg/dL	0.92 (0.58, 1.46)		0.72
Creatinine	$\Delta = 0.5$ μmol/mL	1.29 (0.80, 2.09)		0.29
Hemoglobin	$\Delta = 1.25$ g/dL	1.16 (0.83, 1.60)		0.39
CD34 Cells	$\Delta = 2 \times 10^6$ cells	1.14 (0.84, 1.54)		0.40
Prior Hospitalization?	No Prior Hosp.	3.12 (1.60, 6.05)		<0.001

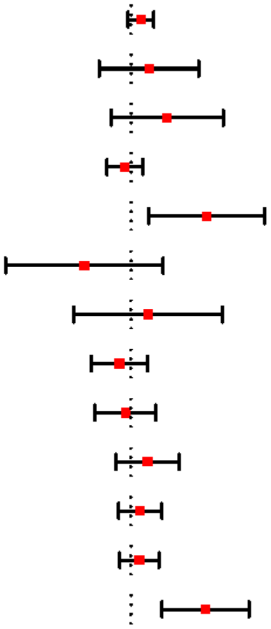

Supplement: Supplementary file 1 [file jcm-11-01640-s001.zip › jcm-1566754-supplementary.pdf]
